# Renal Diagnosis in Medicine and Surgery

**Published:** 1914-06

**Authors:** 


**Renal Diagnosis in Medicine and Surgery.** By Dr. VICTOR BLUM. English Translation by WILFRED B. CHRISTOPHERSON. Pp. viii, 144. 1914. 7s. 6d. net.

This is a good translation of an excellent synopsis of the methods of estimating the functional powers of the kidney.

Beginning with the physiology of the renal functious the author shows how the part played by the glomeruli, convoluted and straight tubules has been proved experimentally.

He then considers the various derangements of the above and the resulting incompetence assignable to each.

This is followed by an account of the various modern functional tests by which the power and reserve power of the kidneys can be estimated, as a preliminary to operative procedure.

In the final chapter definite cases illustrate the significance of the findings of functional diagnosis.

